# Partial hypoglossal-facial neurorrhaphy: a surgical technique

**DOI:** 10.3171/2022.10.FOCVID2290

**Published:** 2023-01-01

**Authors:** Ujwal Yeole, Lissandro Ferrucci, Horacio Murga, Mariano Socolovsky

**Affiliations:** 1 Department of Neurosurgery, Fortis Hospitals, Mumbai, Maharashtra, India;; Departments of 2 Neurosurgery and; 3 ENT, Austral University Hospital, Pilar Provincia de Buenos Aires, Argentina; and; 4 Department of Neurosurgery, Hospital De Clinicas, University of Buenos Aires School of Medicine, Buenos Aires, Argentina

**Keywords:** facial palsy, facial reanimation, hypoglossal-facial neurorrhaphy, nerve coaptation

## Abstract

This is a video demonstration of hemihypoglossal-facial neurorrhaphy. Any irreversible facial palsy more than House-Brackmann grade II is aesthetically problematic. Also, the functional and psychological impact associated with a higher grade of facial palsy is even worse.

In this video, the procedure is demonstrated in a female patient with irreversible grade VI facial palsy and corneal opacity due to exposure keratitis 1 year following surgical excision of cerebellopontine angle (CPA) schwannoma. Postoperative outcomes are shown in another patient who underwent a similar procedure for establishing outcomes. The nasolabial fold and eye closure symmetry can be achieved with minimal or no hypoglossal morbidity.

The video can be found here: https://stream.cadmore.media/r10.3171/2022.10.FOCVID2290

## Transcript

### 0:25

Hello. In this video, we will show you the technical nuances of a surgical nerve transfer involving partial hypoglossal-facial nerve neurorrhaphy for the treatment of chronic facial nerve palsy.

### 0:36

This is a 45-year-old lady who, in June 2021, underwent surgery for total resection of a right CPA schwannoma but was observed to have House-Brackmann grade VI facial nerve (cranial nerve VII) palsy postoperatively, as well as complete loss of eighth cranial nerve function. Over the first postoperative year, she experienced no facial nerve recovery and was referred to us. This is the patient just prior to undergoing facial nerve repair, still exhibiting complete House-Brackmann grade VI facial nerve palsy.

Spontaneous recovery can occur up to 1 year postsurgical excision of schwannoma if intraoperative physiological integrity is confirmed. When anatomical integrity is lost during the removal of a schwannoma, facial reanimation can be considered at 3 months. Otherwise, waiting up to 9–12 months is acceptable.

### 1:37

The surgical procedure involved a retroauricular mastoidectomy, followed by facial nerve and partial hypoglossal nerve neurorrhaphy. This approach has been reported in numerous publications, first described roughly 2 decades ago.^1^ Previously, complete or half of the hypoglossal nerve was used for neurorrhaphy, which leads to long-term hypoglossal morbidity. Our technique of dissecting the epineurium of the facial nerve reduces the width of the hypoglossal nerve required to anastomose (less than half), preventing tongue morbidity associated with older techniques. It consists of drilling out the mastoid segment of the facial nerve and sectioning the nerve as proximal as possible to permit nerve transfer of part of the 12th cranial (hypoglossal) nerve to it.

### 2:31

This is the typical retroauricular approach, where you can see the continuation of the incision 1–2 cm behind the angle of the mandible at the neck.

### 2:42

The procedure involves several steps. The first step involves drilling into the mastoid bone to free the mastoid segment of the facial nerve after creating a retroauricular incision. Usually, this can be performed either by an audiologist or by a neurosurgeon with both training and experience in mastoid bone drilling. We begin by opening the antrum and middle ear cavity, recalling that this patient had already lost the eighth cranial nerve (auditory) function; consequently, opening the middle ear cavity carries no auditory risk. Once we obtain an adequate length of the facial nerve, which includes opening the third part and most distal segment of the second portion of the facial nerve, we elevate the nerve from the bone. To achieve this, we need to completely free the nerve at its mastoid segment, which requires severing the nerves of the chorda tympani. Once the nerve is free, it can be cut as proximal as possible and lifted from the bone.

### 3:53

The second step of the procedure is neck dissection to access the hypoglossal (12th cranial) nerve. To do this, we extend our incision into the neck, dissecting the neck until the posterior part of the digastric muscle is revealed. Below the digastric muscle, we will find the hypoglossal nerve, which can be identified using nerve stimulation. At this point, the ansa hypoglossi (also called the ansa cervicalis)—a loop of nerves part of the cervical plexus—can be identified and dissected. Stimulation of the ansa hypoglossi will cause contraction of the ipsilateral hemitongue, which can be felt by inserting one’s finger into the patient’s mouth.

### 4:36

The procedure’s third stage entails dissecting the extracranial facial nerve, opening the stylomastoid foramen, and redirecting the mastoid segment of the facial nerve downward. This is one of the most critical parts of the procedure. While doing this, you will see the parotid gland and extracranial facial nerve. Adherence of the facial nerve to the stylomastoid foramen is very strong. Both segments of the facial nerve must be dissected, extracranially and intracranially, without hurting the nerve itself. To do this, several branches of the nerve must be cut, including, for instance, the branch to the digastric muscle, which, of course, already is denervated by facial palsy, so no additional morbidity is sustained. Once we free the nerve of the stylomastoid foramen, we redirect the nerve downward toward the hypoglossal nerve. The greater auricular nerves can be preserved.

### 5:43

The fourth step involves working on the facial nerve, which begins with a microsurgical dissection of the epineurium. Typically, within the epineurium, the facial nerve diminishes to half its former width, which is crucial to obtaining a narrower facial nerve and thereby needing to harvest less of the hypoglossal nerve.

### 6:07

The fifth surgical step entails opening the epineurium and dissecting the fascicles of the hypoglossal nerve. Recall that, at this point, the hypoglossal nerve is either mono- or oligofascicular, so there is usually not much connective tissue. Again employing microsurgical techniques, we separate just the part that we need to completely cover the surface of the facial nerve, which is usually between one-third to, at most, one-half of the hypoglossal nerve.

### 6:40

Once this is done, stitches can be placed. Remember that because there is no longer any epineurium, the stitches must be as superficial as possible. Only with two to, at most, three stitches can a perfect match between the donor and the receptor nerve be obtained while ensuring that all the stitches are superficial enough that the fascicles are not injured. Gelfoam and fibrin glue are used to strengthen the sutures. In our experience, few stitches along with the application of glue give better results (more than 90% of success, as published before).^1^ We don’t have any personal experience with the use of conduit wraps or glue alone. We don’t disapprove these techniques as possible methods of neurorrhaphy. Having said that, we believe that the use of glue without suturing might eventually increase the risk of dehiscence of the nerve suture. The procedure is completed by covering the mastoid bone with Gelfoam and closing the skin in the usual manner. Postoperatively, rehabilitation begins using facial physiotherapy initially for a few months to prevent facial muscle atrophy, and once muscle contraction reappears, we add electrostimulation along with physiotherapy.

### 7:48

Here, the typical surgical outcome of this procedure is shown in another patient who sustained complete palsy that had persisted 9 months after the complete resection of a vestibular schwannoma. Eighteen months after undergoing the above-described surgical procedure, observe how the patient has good symmetry of both the mouth and remaining face and good eye closure. She was very satisfied with the surgical outcome.

These are some of the references we used while creating this video.^1–4^

We want to thank you all for your attention.

## Disclosures

The authors report no conflict of interest concerning the materials or methods used in this study or the findings specified in this publication.

## Author Contributions

Primary surgeon: Socolovsky, Murga. Assistant surgeon: Yeole, Ferrucci. Editing and drafting the video and abstract: Socolovsky, Yeole. Critically revising the work: Ferrucci. Reviewed submitted version of the work: Socolovsky. Approved the final version of the work on behalf of all authors: Socolovsky.

## Supplemental Information

### Patient Informed Consent

The necessary patient informed consent was obtained in this study.
